# Percutaneous kyphoplasty combined with pediculoplasty for the surgical treatment of osteoporotic thoracolumbar burst fractures

**DOI:** 10.1186/s13018-024-04562-w

**Published:** 2024-01-22

**Authors:** Changming Xiao, Haozhong Wang, Yang Lei, Mingzhong Xie, Sen Li

**Affiliations:** Spinal Surgery Department, The Affiliated Traditional Chinese Medicine Hospital, Southwest Medical University, Luzhou, 646000 Sichuan China

**Keywords:** Osteoporotic thoracolumbar burst fracture, OVCFs, Simple PKP, PKCPP, Pediculoplasty

## Abstract

**Objective:**

This study introduces a minimally invasive technique for efficient three-column reconstruction, augmentation, and stabilization of osteoporotic thoracolumbar burst fractures (OTLBFs).

**Methods:**

Sixty-eight patients with OTLBFs and no neurological deficits were included from July 2019 to September 2020. The patients were divided into two groups: the simple percutaneous kyphoplasty (PKP) group (*n* = 32) and the percutaneous kyphoplasty combined with pediculoplasty (PKCPP) group (*n* = 36). The clinical and radiological outcomes were assessed during a minimum 1-year follow-up period. Clinical outcomes were assessed via the visual analog scale (VAS) and modified MacNab grading criteria. The radiological outcomes included the Cobb angle (CA), anterior wall height (AWH), and posterior wall height (PWH). The surgery duration, postoperative analgesic dosage, length of hospital stay, and complications were recorded.

**Results:**

Surgery duration was not significantly different between the two groups (*P* > 0.05). The PKCPP group had a lower analgesic dosage and shorter hospital stay (*P* < 0.05). Postoperatively, the PKCPP group exhibited better VAS scores and modified MacNab scale scores (*P* < 0.05), but the differences at the last follow-up assessment were not significant (*P* > 0.05). Postoperative CA, AWH, and PWH correction were not significantly different on the first postoperative day (*P* > 0.05). However, the PKCPP group had significantly less CA and PWH loss of correction at the last follow-up visit (*P* < 0.05). The PKCPP group had significantly fewer complications (*P* < 0.05).

**Conclusions:**

The PKCPP technique complements simple PKP for OTLBFs. It quickly relieves pain, maintains the vertebral body height and Cobb angle, ensures cement stabilization, and offers more stable three-column support.

## Introduction

An estimated 1.4 million osteoporotic vertebral compression fractures (OVCFs) occur every year worldwide [1, 2]. Owing to the proportion of aging adults increasing worldwide and the associated loss in bone mass, the prevalence of osteoporosis will continue to increase. OVCFs are associated with significant clinical problems, such as persistent severe pain, height loss, kyphotic deformity, reduced pulmonary function, reduced mobility, and increased mortality [3, 4]. The majority of OVCFs occur in the thoracolumbar region [5, 6], and burst fractures accompany approximately 50% of osteoporotic thoracolumbar fractures [7]. However, OTLBFs commonly accompany fractures of the middle column and posterior wall, resulting in severe disruption of spinal stability. Although surgery has been accepted as a main treatment method for OTLBFs [8–10], the optimal surgical strategy has remained unknown.

Percutaneous kyphoplasty (PKP) is a safe and effective method for managing OVCFs; it can effectively relieve pain, restore vertebral height, and augment the anterior column. Thus, PKP is often the first treatment choice for OVCFs [11–13]. However, when PKP is used to treat OTLBFs, there are several disadvantages, such as cement displacement, posterior wall retropulsion, further collapse, kyphosis, and recurrent fracture [14–21]. It is well established that conventional stand-alone pedicle screw fixation or other posterior stabilization strategies may fail more often in older osteoporotic patients because of the destruction of the bone microstructure and increased bone fragility [22–25].

Recently, hybrid stabilization has been viewed as an acceptable therapeutic strategy for unstable OTLBFs and has been associated with good clinical outcomes in most patients [26–29]. The combination of cement augmentation and additional posterior pedicle screw fixation helps to stabilize fractures, thereby preventing severe malalignment. Unfortunately, there are several risks, such as neurological problems, dural laceration, screw and rod breakage, screw loosening, and loss of correction, due to implant failure associated with the use of pedicle screws for OTLBFs [29–34]. Moreover, the general medical condition and bone mass of elderly patients are often poor. Additionally, internal fixation surgery is associated with an increased of the risk of major trauma due to the use of general anesthetics, increases the duration of surgery, lengthens hospital stays, and requires additional bed rest, all of which are not conducive to patient postoperative recovery.

Some scholars believe that treating Kummell's disease by combining percutaneous vertebroplasty (PVP) and bilateral pedicle cement augmentation effectively enhances the stability of the cement block within the vertebrae, prevents displacement of the intravertebral cement, and may reduce postoperative pain [35]. Our team modified the PKP technique by combining percutaneous kyphoplasty with pediculoplasty (PKCPP) to address these concerns and to better manage OTLBFs. Our approach replaces posterior pedicle screw fixation with pedicle bone cement augmentation and bridges the bone cement in both the pedicle and vertebral body. Additionally, this approach increases the contact surface of the bone cement and bone trabecula closely located to the bone cement and surrounding bone tissues, thus increasing the stability of the fractured vertebrae. This approach not only maintains the minimal invasive advantages of PKP but also enhances the stability of the fractured vertebrae, prevents bone cement displacement, and decreases the incidence of vertebral recollapse and refracture. PKCPP may be viewed as a potential novel technique for treating OTLBFs. Hence, a retrospective study comparing a simple PKP group with a PKCPP group was conducted.

## Materials and methods

### Patient population

**Fig. 1:**
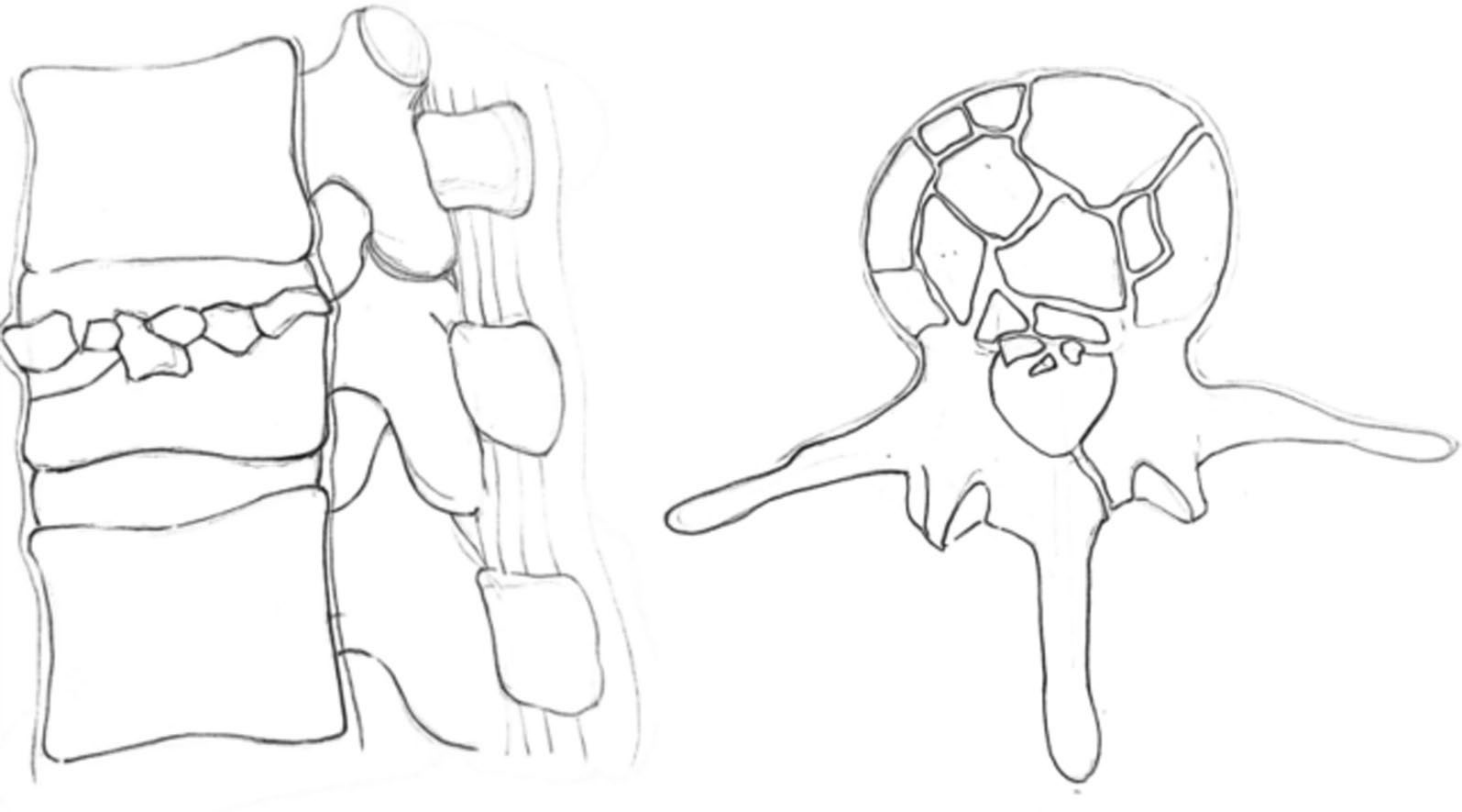
Type A3 fractures are vertebral fractures affecting a single endplate with any involvement of the posterior vertebral wall and spinal canal [36]

From July 2019 to September 2020, 68 patients with OTLBFs (AO classification [36], type A3; Fig. 1) and no neurological deficits were recruited for this study. Thirty-two patients underwent simple PKP (simple PKP group), and 36 patients underwent PKCPP (PKCPP group). The radiological tests performed before surgery included standard anteroposterior and lateral radiographs, computer tomography (CT) scans with axial and sagittal reconstruction, and magnetic resonance imaging (MRI) sequences. The demographic characteristics of the patients are shown in Table 1. All procedures involved bilateral punctures performed by the same group of physicians. The simple PKP or PKCPP instruments were obtained from the Shandong WEGO Company of China, and the polymethylmethacrylate (PMMA) bone cement was obtained from the Heraeus Medical GmbH Company of Germany.

**Table 1:** Preoperative general conditions, intraoperative surgical parameters and postoperative treatment course in the two groups

	Simple PKP group	PKCPP group	*P* value
Age (years, mean ± SD)	73.5 ± 6.1	74.5 ± 5.3	0.523
Gender (M/F)	10/22	13/23	0.799
Fractured level	T11 (6), T12 (12), L1 (10), L2 (4)	T11 (5), T12 (15), L1 (12), L2 (4)	0.946
BMD (*T*-score, mean ± SD)	− 3.93 ± 0.83	− 3.75 ± 0.69	0.149
Cement volume (ml, mean ± SD)	4.03 ± 1.15	4.50 ± 1.26	0.618
**Surgery duration (min, mean ± SD)**	**47.0 ± 10.5**	**52.4 ± 9.4**	**0.332**
Ibuprofen (units, mean ± SD)	8.04 ± 2.07	4.26 ± 1.31	0.012
Hospital stay (days, mean ± SD)	4.69 ± 1.73	2.25 ± 1.05	0.021
Complications			
Cement displacement	2/32 (6.25%)	0/36 (0.00%)	0.218
Cement leakage	4/32 (12.50%)	5/36 (13.89%)	1.000
Refracture	3/32 (9.38%)	0/36 (0.00%)	0.099
Severe recollapse	5/32 (15.62%)	1/36 (2.78%)	0.092
Total	14/32 (43.75%)	6/36 (I6.67%)	0.018

Bold text shows the significant statistical difference

*SD* standard deviation, *BMD* bone mineral density

The inclusion criteria:

1. older than 65 years;
2. chronic back pain or difficulty turning over after conservative therapy;
3. fresh OTLBF (AO classification, type A3) on MRI or CT;
4. no neurological deficits;
5. BMD, *T* value ≤ − 2.5 SD.

The exclusion criteria:

1. Pathologic fracture;
2. cannot tolerate surgery;
3. multilevel osteoporotic vertebral fractures;
4. suspected infection of the vertebra or tissues around the surgical area;
5. BMD, *T* value > − 2.5 SD;
6. blood coagulation dysfunction.

### Surgical procedure

The detailed procedures for percutaneous kyphoplasty (PKP) aligned with previous descriptions of conventional PKP techniques [37, 38]. Fluoroscopy guidance was used throughout the procedure for monitoring.

### Simple PKP group

**Fig. 2:**
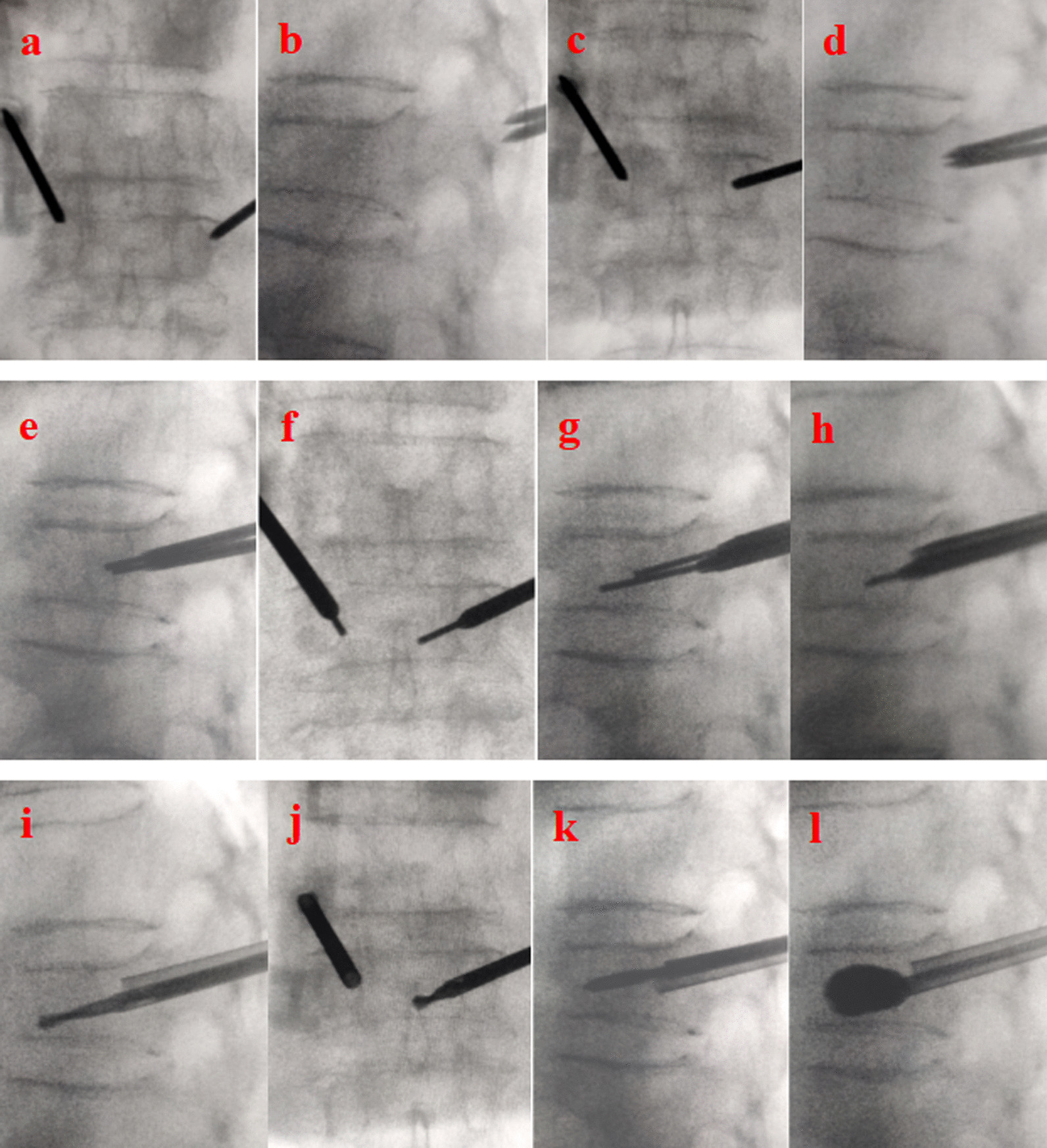
Routine PKP surgery before bone cement injection. **a**–**d** Implantation of the puncture needles; **e**–**h** the working cannulas were established as follows: **i**–**k** The bilateral operating space for the balloon was established; **l** the balloon was inflated, and the vertebral body height was recovered

Under local anesthesia, bilateral transpedicular puncture was performed. The drill was inserted through the working cannulas to create space for the balloon. Subsequently, bilateral balloons were inserted into the vertebral body (VB) through the working tunnel. Balloons were gently implanted to restore the height of the infected vertebra and then deflated after the superior endplate was elevated. After the balloon was removed, the debris from the gelatine sponge was pushed into the anterior one-fourth of the VB through the bilateral working cannula [39]. Subsequently, polymethylmethacrylate (PMMA) bone cement was used to fill the preformed hollow spaces. The procedure was immediately stopped if the bone cement spread too close to the posterior wall or if any signs of leakage were observed (Fig. 2).

### PKCPP group

**Fig. 3:**
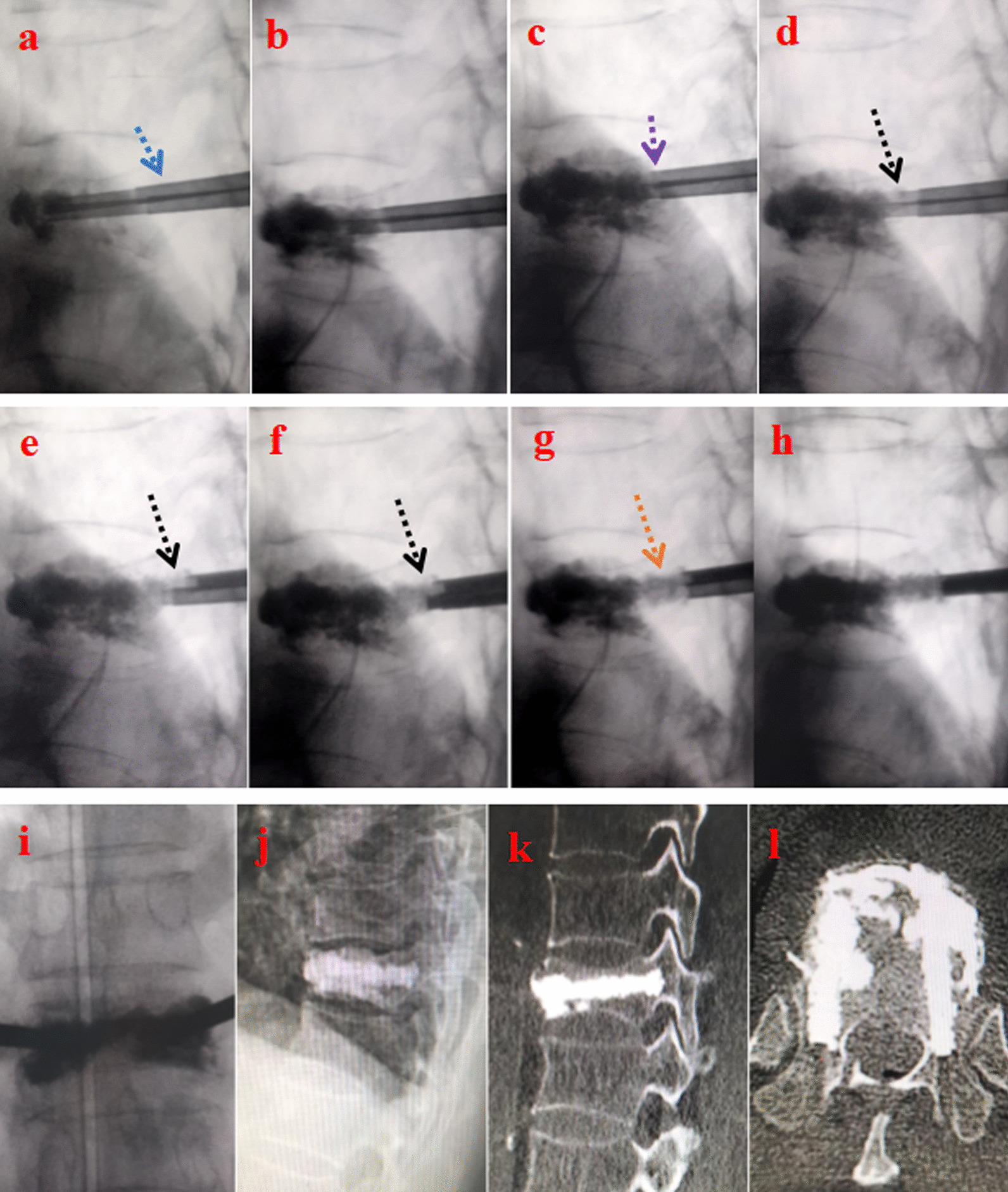
Special operation of the PKCPP surgery, postoperative lateral radiographs, and CT scans. **a** Making the two working cannulas un-overlapped by slightly adjusting C-arm’s position (blue arrow); **b** injecting the bone cement of the anterior two-thirds of vertebral body; **c**–**g** the procedure of pediculoplasty shows that the cement distribution is columnar shape; **h**–**i** intraoperative standard anteroposterior and lateral radiographs show the augmented vertebral body. **j**–**l** Postoperative lateral radiographs and CT scans show that the distribution of bone cement is like two pedicle screws bridging the cement-augmented vertebral body

Compared with simple PKP, bone cement injection was associated with procedural differences. First, it is important to note that the position of the C-arm was fully adjusted so that the two working cannulas did not overlap (Fig. 3a). After this adjustment was made, the PMMA bone cement was injected slowly until the anterior two-thirds (anterior column) of the VB were adequately filled. If the bone cement spread too close to the posterior wall or showed signs of leakage, the injection procedure for the anterior two-thirds of the vertebral body was immediately stopped (Fig. 3b). Next, the bone cement injector was slowly moved backward, while a moderate amount of bone cement was injected until the top of the injector overlapped with the top of the working cannula (Fig. 3c). When the bone cement entered the toothpaste-like consistency, the working cannula and the bone cement injector were slowly moved approximately 3 mm each, and an equal length of bone cement was injected. This process was repeated by continuously moving the working cannula and cement injector backward, with multiple injections of equal lengths of cement until the cannula was withdrawn through the entire pedicle or was close to the initial puncture site (Fig. 3d–f). The bilateral operations were performed simultaneously. It is worth noting that if the cement distribution during pediculoplasty did not resemble a columnar shape (Fig. 3g), waiting until the cement reached the dough stage was recommended. If the cement was moving perpendicular to the pedicle, pediculoplasty was stopped.

All patients in both groups were encouraged to ambulate on the first day after surgery while wearing a protective brace. The brace was required to be worn for at least 1 month. Additionally, all patients in both groups received standard anti-osteoporotic medications (calcium + vitamin D + bisphosphonate) after surgery [40–42].

### Clinical and radiographic evaluation

The surgical duration, postoperative analgesic medication dosage, volume of injected bone cement, incidence of cement leakage, and duration of hospital stay were recorded for each group. Postoperative outcomes were evaluated at 1 day, 1 month, 3 months, and 12 months after surgery using the VAS score and the modified MacNab grading criteria. Any postoperative surgical complications were documented.

Radiological parameters were measured by the same physician before and after surgery using radiographs, CT scans, or MRI scans. The angle between the superior and inferior endplate lines of the adjacent vertebra (CA), AWH, and PWH was measured on lateral radiographs. CT or MRI scans were utilized to assess bone cement leakage, distribution, cement displacement, and vertebral refracture.

### Statistical analysis

The statistical analysis was performed using SPSS software (version 19.0; SPSS, Inc., IL, USA). The Mann–Whitney U test was used to compare age and bone mineral density (BMD) between the two groups. The Chi-square test was used to assess differences in sex distributions and fracture-level ratios. Independent sample *t* tests were also conducted to compare variables such as operative time, postoperative analgesic medication dosage, duration of hospital stay, AWH, PWH, CA, and VAS score between the two groups. Fisher’s exact test was used to compare the rates of complications and the rates of meeting excellent and good criteria in both groups. A *P* value < 0.05 was set to indicate significance.

## Results

Table 1 presents the surgical findings and postoperative treatment course. The mean surgery duration was not significantly different between the two groups (*P* = 0.332). Patients in the simple PKP group required a significantly greater amount of ibuprofen than did those in the PKCPP group (7.75 ± 1.55 units vs. 5.94 ± 1.12 units; *P* = 0.024). Furthermore, the simple PKP group had a significantly longer hospital stay than the PKCPP group (4.69 ± 1.73 days vs. 2.25 ± 1.05 days; *P* = 0.021).

**Fig. 4:**
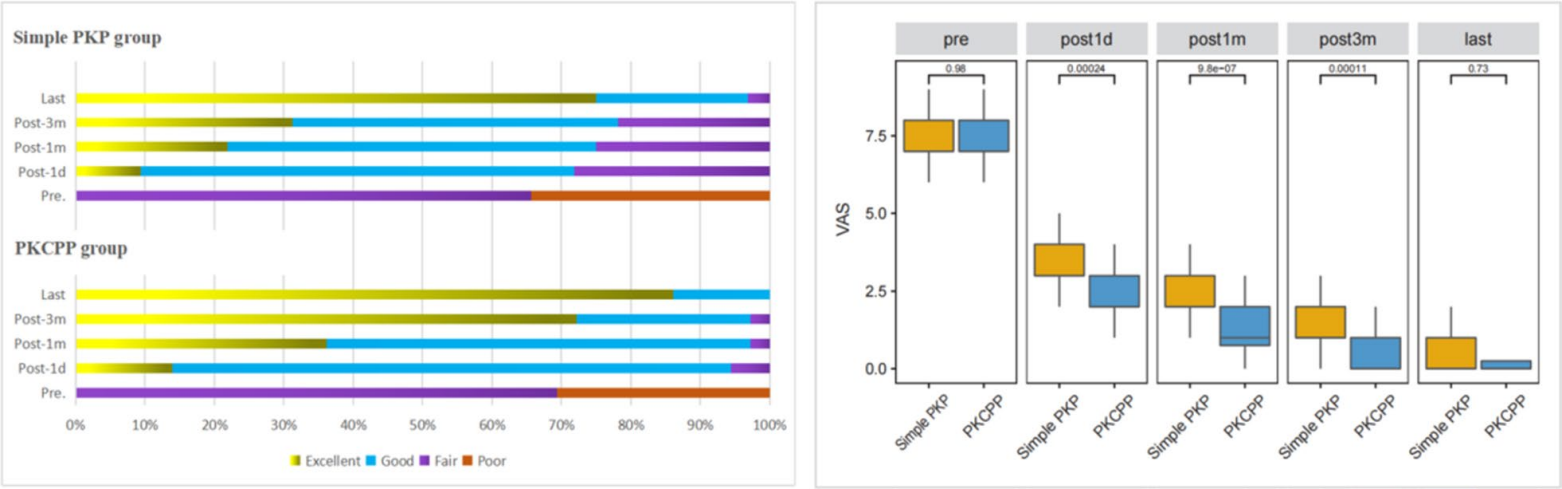
Clinical results, including the MacNab scale score and mean VAS score for back pain in both groups. The PKCPP group had a greater percentage of patients who met the excellent and good criteria than the simple PKP group at 1 day, 1 month, and 3 months after surgery (*P* = 0.019, 0.010, and 0.022, respectively); however, the modified MacNab scale score did not significantly differ between the two groups at the last follow-up visit (*P* = 0.471). The VAS score significantly decreased after surgery in both groups (*P* < 0.05), and the PKCPP group had better VAS scores than the simple PKP group at 1 day, 1 month, and 3 months after surgery (all *P* < 0.001); however, the VAS scores in the two groups were not significantly different at the last follow-up visit (*P* = 0.73)

The VAS score significantly decreased after surgery in both groups (*P* < 0.05). Additionally, at 1 day, 1 month, and 3 months after surgery, the PKCPP group exhibited better VAS scores, with a greater percentage of patients meeting the modified MacNab grading criteria than the simple PKP group (*P* < 0.05). However, at the last follow-up assessment, the VAS score and modified MacNab grade did not significantly differ between the two groups (*P* > 0.05; Fig. 4).

**Fig. 5:**
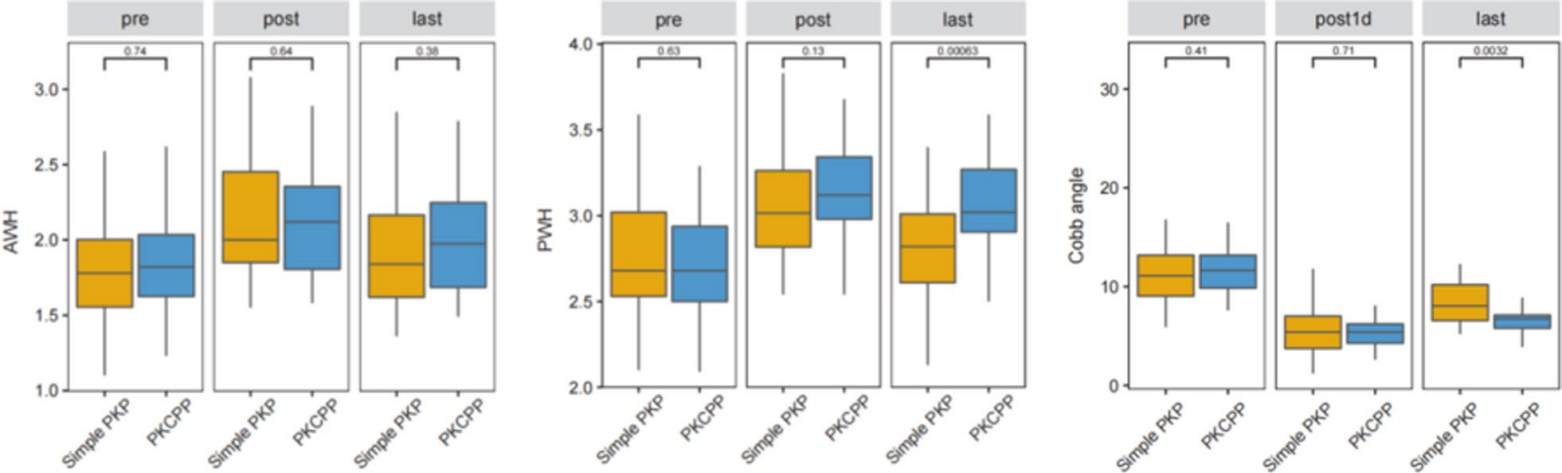
AWH, anterior wall height; PWH, posterior wall height; CA, Cobb angle. AWH and PWH were significantly increased on day 1 after surgery in both groups but were slightly lower at the follow-up assessment. Moreover, the PWH loss of correction in the PKCPP group was significantly less than that in the simple group at the last follow-up visit (*P* < 0.001). The CA was significantly decreased in both groups on day 1 after surgery, but there were no significant differences in the CA (*P* = 0.71). However, the postoperative CA loss at correction in the PKCPP group was significantly less than that in the simple group at the last follow-up visit (*P* < 0.05)

The AWH, PWH, and CA significantly improved at 1 day after surgery in both groups. There was no significant difference in postoperative CA, AWH, or PWH correction between the two groups at 1 day after surgery (*P* = 0.71, 0.64, 0.13). However, at the last follow-up visit, compared to the simple PKP group, the PKCPP group demonstrated significantly less postoperative CA and PWH loss of correction (*P* < 0.05; Fig. 5).

No perioperative nerve damage or infections were observed in either group. CT scans also indicated that there was no evidence of bone cement leakage into the spinal canal through the pedicle in the PKCPP group. The incidence of complications, including cement displacement, cement leakage, recurrent fracture, and severe recollapse, in the PKCPP group was significantly lower than that in the simple PKP group (*P* = 0.018) (Table 1). Two cases of cement displacement occurred in the simple PKP group. One patient declined further surgical intervention but experienced pain relief through analgesic and anti-osteoporosis drug treatment. The other patient underwent revision surgery via a posterior approach along with regular anti-osteoporosis drug therapy.

## Discussion

In 1984, Galibert was first to perform PVP for the treatment of vertebral hemangiomas and report favorable clinical outcomes [43]. After several decades of development, this technique has gradually become the gold standard in the clinical treatment of OVCFs. PVP not only effectively enhances vertebral strength and rigidity but also rapidly alleviates pain and facilitates the return to daily activities. However, OTLBFs often accompany fractures of the middle column and posterior wall, leading to controversy regarding treatment strategies, especially among elderly patients. Van Der Schaaf et al. [35] treated Kummell’s disease by combining bilateral vertebroplasty with bilateral pedicle internal augmentation using bone cement. By connecting the three columns of the spine with bone cement, they achieved favorable therapeutic effects. They believe that this method effectively prevents the displacement of bone cement, as it prevents subsequent posterior vertebral wall impingement into the spinal canal, which could lead to neurological symptoms. The authors speculated that the shaping of the pedicle by bone cement is a key factor in this approach.

The PKCPP surgical technique involves filling the vertebral body with bone cement via a bilateral pedicle puncture approach. Subsequently, the working cannula and bone cement injector are continuously moved dorsally, while an equal length of bone cement is injected. This process is continued until the working cannula and cement injector are completely evacuated from the entire pedicle. This procedure connects the anterior and middle columns of the vertebra as well as the bilateral pedicles by using bone cement to form a unified structure. At present, there are no published reports on this surgical method for the treatment of OTLBF. Therefore, we retrospectively compared the clinical efficacy and imaging parameters between simple PKP and PKCPP. For the treatment of OTLBFs in elderly patients, PKCPP has significant advantages over simple PKP in terms of postoperative back pain relief, cement displacement, posterior wall retropulsion, further vertebral collapse, and recurrent fracture. Postoperative radiographs and CT scans demonstrated good cement distribution and filling of the bone defect. Because PKCPP is equally as minimally invasive as the PKP technique, it does not require new equipment or instruments, can be performed in the same amount of time, and can effectively reduce the incidences of postoperative complications and trauma. Moreover, because the bone cement in both the pedicle and the vertebral body is closely bridged, this approach provides more stable three-column support and adds complementary advantages to the pedicle screw technique and PKP technique. The present study demonstrated that the PKCPP is effective and safe for the treatment of OTLBFs in elderly patients.

In our study, although all patients experienced rapid postoperative pain relief, the PKCPP group had markedly lower VAS scores than the simple PKP group at 1 day, 1 month, and 3 months after surgery; the stabilization of micromotions in the fractured vertebra is hypothesized to be the main mechanism for pain relief after percutaneous vertebral augmentation, and insufficient cement distribution in the fractured area is considered the reason for the increased instability [44, 45]. On the basis of our team’s belief that pediculoplasty during PKCPP surgery can closely connect and fix the fracture pieces of the vertebral body, similar to the action of pedicle screws, the displacement of the fracture area can be reduced, allowing pain to be significantly relieved after surgery. Furthermore, bilateral puncture promotes the effective dispersion of bone cement into the loose bone trabecular space, fully filling the crevice of the fractured vertebra, and better reconstructing the three anterior-middle-posterior columns of the diseased vertebra, thereby confirming its good supporting role.

Although bone cement displacement is a rare complication, some studies have shown that it can cause vertebral collapse, local instability of the spine, and pseudarthrosis, which may lead to intractable pain, aggravation of kyphosis, and even neurological impairment [16, 19–21]. If this occurs, the open posterior or anterior approach or even a combination of anterior and posterior approach revision surgery is usually needed. This revision surgery is not only difficult and risky; it is also difficult for older patients to tolerate, making treatment extremely difficult. The incidence of bone cement displacement in PKP surgery was 6.4% in a previous study [16]. In our study, two cases of bone cement displacement were found in the simple PKP group (2/32, 6.25%); however, no bone cement displacement was found in the PKCPP group, possibly because the PKCPP technique connects with the bone cement both in the pedicle and the vertebral body as a whole. Additionally, increasing the contact surface of the bone cement and bone trabecula effectively joins the bone cement and surrounding bone tissues, increasing the stability of the fractured vertebrae. One study revealed that the tensile strength of the cement–bone interface was inversely proportional to the compliance of the interface and proportional to the cement–bone contact area, and the strength increased with increasing contact area [46]. An interface is formed when PMMA cement is injected into bone cavities, which provides fixation for cemented implants within the bone. This results in a highly variable interaction between the bone and cement with complex morphology and mechanical properties [47]. We also believe that the PKCPP technique can increase the contact area between the bone cement and surrounding bone tissue and that more anchor points can be removed from the bone cement. Another study proposed that sufficient penetration of bone cement into the microstructure of the trabecular bone during PKP surgery could reduce the risk of bone cement displacement [48]. An anterior cortex defect may increase the risk of anterior displacement of the cement under weight-bearing conditions; thus, the integrity of the anterior cortex is also an important consideration when assessing anterior displacement [16]. Considering that, in patients with OTLBFs, the anterior cortex of the vertebral body will rupture to different degrees, excessive reduction may increase the severity of the defect. Therefore, none of the patients in this study underwent postural reduction or manipulative reduction, the vertebral body height did not increase significantly compared with that preoperatively, and the incidence of cement displacement was lower than that reported in previous literature.

Almost all patients had a slight decrease in vertebral height at the follow-up assessment, and some studies have suggested that an asymmetrical cement distribution around the fractured area was the main risk factor for recollapse of the augmented vertebral bodies [31, 49, 50]. A biomechanical study indicated that symmetrical cement distribution around the fractured area may provide better structural support and decrease the incidence of recollapse [44]. Another study suggested that fibrotic wall formation around a PMMA mass may induce micromotion and future instability, which may induce recollapse of the augmented vertebral body [32]. In this study, progressive vertebral collapse and aggravation of kyphosis were found in both groups, but the rates of CA and PWH loss of correction in the PKCPP group were significantly lower than those in the simple PKP group at the last follow-up visit. Additionally, bilateral puncture allows for a more symmetrical cement distribution and more adequate cement filling. Moreover, the cement-augmented pedicle may play the role of a pedicle screw, which shares part of the load of the anterior column, effectively reducing the degree of collapse and kyphosis deformity. More importantly, due to the connection of the bone cement both in the pedicle and in the vertebral body, this approach is similar to the process of forming a complete vertebral body with greater strength and stiffness, which is effective at preventing vertebral collapse and kyphosis.

The literature-reported incidence rates of adjacent vertebral fractures after PKP surgery vary from 6.5 to 25% [51]. Vertebral refractures often cause worsening of back pain and kyphosis deformity, which impact patient quality of life. Lavelle and Cheney et al. reported a 10% incidence of refracture of the augmented vertebra after PKP [14, 18]. During the follow-up period, 3 patients in the simple PKP group experienced recurrent fractures (3/32, 9.38%), and there were no patients in the PKCPP group with recurrent fractures. However, some studies have indicated that the main cause of refracture is not PVP or PKP surgery but rather significant osteoporosis and an imbalance of mechanical distribution between vertebral bodies [52]. We believe that the symmetrical cement distribution and adequate cement filling in the PKCPP group increase the uniformity and effectiveness of the mechanical distribution, thereby reducing the risk of recurrent fractures. Moreover, an increase in the forward bending moment requires an increase in the counterbalancing posterior force from the musculature and ligaments, which can cause paraspinal muscle fatigue and chronic back pain in patients with osteoporotic spinal kyphotic deformity [51]. Therefore, we attach great importance to standardized anti-osteoporosis treatment and back muscle function training after surgery.

Bone cement leakage is the most common complication of PKP and can cause severe spinal cord nerve damage, pulmonary embolism, and intracardiac embolism and diminish patient quality of life and safety. It has been previously reported that the bone cement leakage rate in patients with OTLBFs undergoing PKP was 23.1–45.4% [13, 28, 53]. In the present study, 4 cases (12.5%) of cement leakage were observed in the simple PKP group, and 5 cases (13.89%) of cement leakage were observed in the PKCPP group, suggesting that the leakage rate in the PKCPP group was acceptable. Furthermore, no patients in either of the two groups exhibited any of the aforementioned neurological or other systematic complications. Our team believes that the above results may be due to the following reasons. First, all patients underwent bilateral punctures, resulting in less pressure in the vertebral body [48]. Second, the degree of reduction in the anterior column was low, and the fissures in the fractured vertebral body did not increase significantly. Third, all patients were filled with gelatine sponge debris before bone cement injection. Finally, the injection of cement was stopped immediately when the bone cement spread too close to the posterior wall of the vertebral body or appeared to leak.

As a minimally invasive surgery, the PKCPP can promote a rapid return to activities of daily living and may prevent complications in elderly patients who remain in bed for a long period of time. PKCPP effectively reduced the incidence of postoperative complications and may be preferable when considering the length of postoperative hospital stay, analgesic dosage, further collapse, kyphosis, and postoperative VAS score. Notably, postoperative regular use of calcium and vitamin D combined with bisphosphonates is a key factor for successful treatment [54–57]. Because the sample size and short follow-up period were limitations, long-term follow-up of this technique needs to be explored in a future study with a larger sample size.

## Conclusions

As a supplement to the simple PKP surgical technique for OTLBFs, the PKCPP technique should be considered not only as a rapid pain-relieving procedure but also as an effective method for maintaining vertebral body height, Cobb angle, and stabilization of bone cement and for providing more stable three-column support.

## Abbreviations

PKCPP: Percutaneous kyphoplasty combined with pediculoplasty
OTLBF: Osteoporotic thoracolumbar burst fracture
VAS: Visual analog scale
AWH: Anterior wall height
PWH: Posterior wall height
CA: Cobb angle
OVCFs: Osteoporotic vertebral compression fractures
PKP: Percutaneous kyphoplasty
PVP: Percutaneous vertebroplasty
CT: Computed tomography
MRI: Magnetic resonance imaging
PMMA: Polymethylmethacrylate
BMD: Bone mineral density
